# Global burden of influenza-associated lower respiratory tract infections and hospitalizations among adults: A systematic review and meta-analysis

**DOI:** 10.1371/journal.pmed.1003550

**Published:** 2021-03-01

**Authors:** Kathryn E. Lafond, Rachael M. Porter, Melissa J. Whaley, Zhou Suizan, Zhang Ran, Mohammad Abdul Aleem, Binay Thapa, Borann Sar, Viviana Sotomayor Proschle, Zhibin Peng, Luzhao Feng, Daouda Coulibaly, Edith Nkwembe, Alfredo Olmedo, William Ampofo, Siddhartha Saha, Mandeep Chadha, Amalya Mangiri, Vivi Setiawaty, Sami Sheikh Ali, Sandra S. Chaves, Dinagul Otorbaeva, Onechanh Keosavanh, Majd Saleh, Antonia Ho, Burmaa Alexander, Hicham Oumzil, Kedar Prasad Baral, Q. Sue Huang, Adedeji A. Adebayo, Idris Al-Abaidani, Marta von Horoch, Cheryl Cohen, Stefano Tempia, Vida Mmbaga, Malinee Chittaganpitch, Mariana Casal, Duc Anh Dang, Paula Couto, Harish Nair, Joseph S. Bresee, Sonja J. Olsen, Eduardo Azziz-Baumgartner, J. Pekka Nuorti, Marc-Alain Widdowson, Global Respiratory Hospitalizations–Influenza Proportion Positive (GRIPP) Working Group

**Affiliations:** 1 Influenza Division, US Centers for Disease Control and Prevention, Atlanta, Georgia, United States of America; 2 Health Sciences Unit, Faculty of Social Sciences, Tampere University, Tampere, Finland; 3 US Centers for Disease Control and Prevention, Atlanta, Georgia, United States of America; 4 Influenza Division, US Centers for Disease Control and Prevention, Beijing, China; 5 Program for Emerging Infections, Infectious Diseases Division, icddr,b, Dhaka, Bangladesh; 6 Royal Centre for Disease Control, Thimphu, Bhutan; 7 Centers for Disease Control and Prevention, Phnom Penh, Cambodia; 8 Department of Epidemiology, Ministerio de Salud, Santiago, Chile; 9 Division of Infectious Diseases, Chinese Center for Disease Control and Prevention, Beijing, China; 10 School of Population Medicine & Public Health, Chinese Academy of Medical Sciences/Peking Union Medical College, Beijing, China; 11 Institut National d’Hygiène Publique, Abidjan, Côte d’Ivoire; 12 Institut National de Recherches Biomédicales, Kinshasa, République Démocratique du Congo; 13 Ministerio de Salud, Quito, Ecuador; 14 Noguchi Memorial Institute for Medical Research, College of Health Sciences, University of Ghana, Accra, Ghana; 15 Influenza Division, US Centers for Disease Control and Prevention, New Delhi, India; 16 National Institute of Virology, Pune, India; 17 US Centers for Disease Control and Prevention, Jakarta, Indonesia; 18 National Institute of Health Research and Development, Jakarta, Indonesia; 19 Ministry of Health, Amman, Jordan; 20 Influenza Division, US Centers for Disease Control and Prevention, Nairobi, Kenya; 21 Department of State Sanitary Epidemiological Surveillance, Bishkek, Kyrgyzstan; 22 National Center for Laboratory and Epidemiology, Vientiane, Lao People’s Democratic Republic; 23 Epidemiological Surveillance Program, Lebanese Ministry of Public Health, Beirut, Lebanon; 24 MRC–University of Glasgow Centre for Virus Research, Glasgow, United Kingdom; 25 Malawi–Liverpool–Wellcome Trust Clinical Research Programme, Blantyre, Malawi; 26 National Centre for Communicable Disease, Ulaanbaatar, Mongolia; 27 Virology Department, Institut National d’Hygiène, Rabat, Morocco; 28 Faculty of Medicine, Microbiology RPU, Mohammed V University, Rabat, Morocco; 29 Patan Academy of Health Sciences, Lalitpur, Nepal; 30 WHO National Influenza Centre, Institute of Environmental Science and Research, Wellington, New Zealand; 31 Nigeria Centre for Disease Control, Federal Ministry of Health, Abuja, Nigeria; 32 Directorate General of Disease Surveillance and Control, Ministry of Health, Muscat, Oman; 33 Ministerio de Salud Publica y Bienestar Social, Asunción, Paraguay; 34 National Institute for Communicable Diseases, Johannesburg, South Africa; 35 MassGenics, Duluth, Georgia, United States of America; 36 School of Public Health, Faculty of Health Sciences, University of the Witwatersrand, Johannesburg, South Africa; 37 Ministry of Health, Dar es Salaam, Tanzania; 38 National Institute of Health, Department of Medical Sciences, Ministry of Public Health, Nonthaburi, Thailand; 39 Arizona Department of Health Services, Phoenix, Arizona, United States of America; 40 National Institute of Hygiene and Epidemiology, Hanoi, Vietnam; 41 Pan American Health Organization, Washington, District of Columbia, United States of America; 42 Centre for Global Health, Usher Institute, University of Edinburgh, Edinburgh, United Kingdom; 43 Finnish Institute for Health and Welfare, Helsinki, Finland; 44 Division of Global Health Protection, US Centers for Disease Control and Prevention, Nairobi, Kenya; 45 Institute of Tropical Medicine, Antwerp, Belgium; Universitair Medisch Centrum Utrecht, NETHERLANDS

## Abstract

**Background:**

Influenza illness burden is substantial, particularly among young children, older adults, and those with underlying conditions. Initiatives are underway to develop better global estimates for influenza-associated hospitalizations and deaths. Knowledge gaps remain regarding the role of influenza viruses in severe respiratory disease and hospitalizations among adults, particularly in lower-income settings.

**Methods and findings:**

We aggregated published data from a systematic review and unpublished data from surveillance platforms to generate global meta-analytic estimates for the proportion of acute respiratory hospitalizations associated with influenza viruses among adults. We searched 9 online databases (Medline, Embase, CINAHL, Cochrane Library, Scopus, Global Health, LILACS, WHOLIS, and CNKI; 1 January 1996–31 December 2016) to identify observational studies of influenza-associated hospitalizations in adults, and assessed eligible papers for bias using a simplified Newcastle–Ottawa scale for observational data. We applied meta-analytic proportions to global estimates of lower respiratory infections (LRIs) and hospitalizations from the Global Burden of Disease study in adults ≥20 years and by age groups (20–64 years and ≥65 years) to obtain the number of influenza-associated LRI episodes and hospitalizations for 2016. Data from 63 sources showed that influenza was associated with 14.1% (95% CI 12.1%–16.5%) of acute respiratory hospitalizations among all adults, with no significant differences by age group. The 63 data sources represent published observational studies (*n =* 28) and unpublished surveillance data (*n =* 35), from all World Health Organization regions (Africa, *n =* 8; Americas, *n =* 11; Eastern Mediterranean, *n =* 7; Europe, *n =* 8; Southeast Asia, *n =* 11; Western Pacific, *n =* 18). Data quality for published data sources was predominantly moderate or high (75%, *n =* 56/75). We estimate 32,126,000 (95% CI 20,484,000–46,129,000) influenza-associated LRI episodes and 5,678,000 (95% CI 3,205,000–9,432,000) LRI hospitalizations occur each year among adults. While adults <65 years contribute most influenza-associated LRI hospitalizations and episodes (3,464,000 [95% CI 1,885,000–5,978,000] LRI hospitalizations and 31,087,000 [95% CI 19,987,000–44,444,000] LRI episodes), hospitalization rates were highest in those ≥65 years (437/100,000 person-years [95% CI 265–612/100,000 person-years]). For this analysis, published articles were limited in their inclusion of stratified testing data by year and age group. Lack of information regarding influenza vaccination of the study population was also a limitation across both types of data sources.

**Conclusions:**

In this meta-analysis, we estimated that influenza viruses are associated with over 5 million hospitalizations worldwide per year. Inclusion of both published and unpublished findings allowed for increased power to generate stratified estimates, and improved representation from lower-income countries. Together, the available data demonstrate the importance of influenza viruses as a cause of severe disease and hospitalizations in younger and older adults worldwide.

## Abstract 2

In this meta-analysis, Kathryn E. Lafond and colleagues estimate the global hospitalisation burden from influenza infections in adults.

## Author summary

**Why was this study done?:**

- Every year, millions of people become infected with influenza viruses, which infect the airways and cause symptoms that include a fever, tiredness and weakness, general aches and pains, sore throat, and cough.
- Most infected individuals recover quickly, but some develop more severe illness such as pneumonia, and may require hospitalization.
- Influenza vaccination can prevent infections and severe disease, but decisions on how to prioritize risk groups, particularly in limited-resource settings, require a robust evidence base, including global, regional, and local data.

**What did the researchers do and find?:**

- We conducted a systematic review to identify published articles on influenza-associated respiratory disease in hospitalized adults.
- We then aggregated the data from these articles with data collected by hospital-based influenza surveillance, to calculate a pooled estimate of the proportion of adults hospitalized with respiratory disease who are positive for influenza.
- Through this approach, we estimated that influenza was associated with 14% of acute respiratory hospitalizations among adults worldwide.
- This equates to over 5 million influenza-associated hospitalizations per year, with the highest rates among those 65 years and older.

**What do these findings mean?:**

- Estimating the disease burden of influenza allows researchers and policymakers to better understand its global impact across different target groups and allows comparisons with other causes of morbidity and mortality.
- As global populations age, the importance of vaccinating adults, particularly older adults, is likely to increase.

## Introduction

Influenza viruses contribute substantially to respiratory disease worldwide, including severe lower respiratory tract illnesses, hospitalizations, and deaths. The highest rates of severe influenza-associated disease and hospitalization are typically reported among those at the extremes of age [1–4] and those with underlying health conditions [5]. To reduce the burden of influenza, the World Health Organization (WHO) Strategic Advisory Group of Experts on Immunization (SAGE) recommends that countries initiating or expanding an influenza vaccination program prioritize pregnant women, children aged 6–59 months, adults ≥65 years, individuals with high-risk conditions, and healthcare workers [6]. During the COVID-19 pandemic, SAGE has also released interim recommendations prioritizing healthcare workers and older adults for influenza vaccination [7].

Global influenza burden estimates generated to date include all-age estimates of hospitalizations and mortality, as well as age-specific estimates for pediatric hospitalizations [8–10]. Important gaps in knowledge remain about the burden of influenza-associated severe respiratory disease and hospitalizations in adults, particularly in lower-resource settings, because of the dearth of denominators for incidence calculations [11]. The objective of these analyses was to generate a global estimate of the annual number of respiratory hospitalizations and lower respiratory infections (LRIs) associated with influenza viruses among adults ≥20 years worldwide, utilizing published findings and unpublished laboratory-confirmed data from a global network of influenza surveillance partners.

## Methods

### Research objective and data sources

Our a priori research objective was to estimate the proportion of adult respiratory hospitalizations that tested positive for influenza (“proportion positive”) as a summary statistic. We generated this estimate using 2 sources of data: a systematic review of the literature and unpublished respiratory inpatient surveillance data from a global working group of partners. We then applied the summary meta-analytic proportion positive to global estimates of adult LRI episodes and hospitalizations available for adults ≥20 years.

### Systematic literature review: Search strategy and selection criteria

We searched 9 online databases (Medline, Embase, CINAHL, Cochrane Library, Scopus, Global Health, LILACS, WHOLIS, and CNKI) for articles published in any language from 1 January 1996 to 31 December 2016 to identify studies of influenza-associated hospitalizations in adults. Keywords included “influenza” and terms related to hospitalization (e.g., “hospital*” and “inpatient”) (S1 Table). Titles and abstracts of all articles identified were screened for inclusion in the analysis by a member of the review team (KEL, RMP, MJW, EAB, MAW, J. Kile) with support from native language speakers as needed. Chinese-language articles were screened by native Mandarin speakers (ZS and ZR). A random subset (25%) of articles were screened by a second reviewer for validation. An article was included for full-text review if at least 1 reviewer flagged it as potentially eligible. The inclusion criteria were (1) an original study on human participants; (2) laboratory testing for both influenza A and B viruses, with description of diagnostic methods used; (3) a minimum of 12 months’ continuous data; (4) a specified case definition or other clear criteria for systematic specimen collection and testing; (5) inclusion of hospitalized patients; (6) inclusion of the number of cases from whom clinical specimens were collected and the number found positive; and (7) a minimum of 50 adults ≥18 years of age (or an adult population, as described by authors) tested for influenza across the duration of the study.

We excluded studies that described nosocomial influenza infections or included data from calendar year 2009 (the initial wave of influenza A(H1N1)pdm09 virus circulation) in a manner that could not be separated from data from other years, since both influenza epidemiology and testing practices were atypical during this period. Although the influenza A(H1N1)pdm09 pandemic period continued into 2010, this was often second-wave circulation and was not excluded from the analysis. For titles and abstracts meeting the inclusion criteria, full-text articles were assessed independently by 2 members of the review team. Any discrepant decisions on full-text articles were first discussed between the 2 reviewers; those that could not be resolved (*n =* 2) were adjudicated by a third reviewer (MAW).

From each eligible article, we abstracted the following data elements: (1) the total number of inpatients tested and total positive for influenza by age group and influenza type and subtype where available, (2) location and timeframe of data collection, (3) the case definition and diagnostic tests used, (4) the proportion of the study population with any underlying conditions (as defined by the paper), and (5) influenza vaccination coverage in the study population. We recorded whether the study population was a “special population,” defined as a selection of participants not representative of all inpatients (e.g., intensive care unit patients or those with chronic obstructive pulmonary disease [COPD]). WHO region, World Bank income category (low, lower-middle, upper-middle, or high income), and pneumococcal conjugate vaccine (PCV) introduction status were assigned for each dataset, based on the country and start year of data collection [12,13]. Quality of evidence for each published paper was evaluated on a simplified Newcastle–Ottawa scale, as described previously [8,14]. Key variables (number tested and positive, laboratory method used, and quality score) were abstracted by 2 independent reviewers. We did not preregister the protocol for this systematic review. A full list of eligible published articles with key abstracted data, and a PRISMA checklist for systematic reviews [15], are provided in S2 Table and S1 PRISMA Checklist.

### GRIPP Working Group

To supplement data from published studies, we established a working group, the Global Respiratory Hospitalizations–Influenza Proportion Positive (GRIPP) Working Group, which compiled recent, typically unpublished data through 2016 from surveillance platforms conducting systematic inpatient surveillance for influenza. Eligible surveillance platforms included those that conducted systematic year-round inpatient testing using any acute respiratory case definition (e.g., severe acute respiratory infection [SARI] or community-acquired pneumonia [CAP]) for at least 12 consecutive months and that tested at least 50 adult inpatients over the duration of the study. A minimum of 50 samples was selected to reduce within-study variance and remove undue influence of very small studies. We contacted 73 partners, of whom 37 (51%) had eligible adult data and agreed to participate in the GRIPP Working Group. We collected the following data in a standardized form: the number of persons tested and positive for influenza by calendar year and age group (18–64 years and ≥65 years), the total number of inpatient sites, case definition used, and any participant selection procedures. Patient comorbid conditions and influenza vaccination status were not frequently reported in partner surveillance platforms, and were not collected on the standardized form. If surveillance data were also represented in a paper identified through the systematic review, the working group dataset was used, and the published article was excluded as a duplicate source. Working group data were not scored for quality in the same manner as published articles, but were reviewed for completeness and internal consistency of primary results. Influenza virus detections were reported by type and A subtypes where available; however, as influenza B virus lineage testing was rarely conducted during the study period, it was not included in the current analyses.

### Statistical analysis

We explored all data from both published and unpublished sources in the univariate analysis of median proportion positive by categorical variables (such as diagnostic test used, case definition, WHO region, and country income classification). We tested for differences in frequencies across groups using chi-squared. Since the proportion of respiratory samples testing positive for influenza was not normally distributed, we estimated median values and interquartile ranges (IQRs), and used Kruskal–Wallis non-parametric ANOVA to compare the summary proportion positive per dataset/study across groups. We used a *p*-value of <0.05 as our threshold for significance.

To minimize variability observed among data sources in the univariate analysis, we limited the meta-analysis to data derived from gold-standard reverse transcription PCR (RT-PCR) diagnostic testing and excluded all studies of special populations to estimate the proportion of adult respiratory hospitalizations positive for influenza. To derive meta-analytic estimates of proportion positive, we used a log-binomial mixed model with a random effect for data source that allowed for inclusion of multiple years of data from a single data source [8]. *I*^2^ was calculated as variance across datasets as a percentage of total variance in each model. We generated overall, age-stratified (20–64 and ≥65 years), and influenza A and B virus-specific proportion positive estimates. Virus type-specific models were restricted to datasets with at least 1 type-specific positive sample.

Overall and age-specific meta-analytic estimates of proportion positive were multiplied by LRI hospitalization and LRI episode estimates for adults ≥20 years, which were from the Global Burden of Disease (GBD) 2017 study [10], obtained for calendar year 2016 for this analysis. The GBD study models the incidence of LRI episodes and hospitalizations at a global and regional level, by age group, using a Bayesian meta-regression tool. Confidence intervals for our multiplied estimates of the number of influenza-associated events were created through a Monte Carlo resampling process that assumed normal distribution for denominators and binomial distributions for the modeled proportion positive estimates, using 95% confidence intervals as lower and upper bounds, with 5,000 iterations. We calculated rates by dividing the above estimates by the 2016 global population [16]. Post hoc sensitivity analyses were conducted to explore patterns in heterogeneity (*I*^2^) of the pooled proportion positive and percent change in the estimated number of hospitalizations by stratifying for key covariates. All statistical analyses were conducted in R version 3.5.2, with package “lme4” for mixed models [17,18].

Ethical approval was not required for this study.

## Results

**Fig 1:**
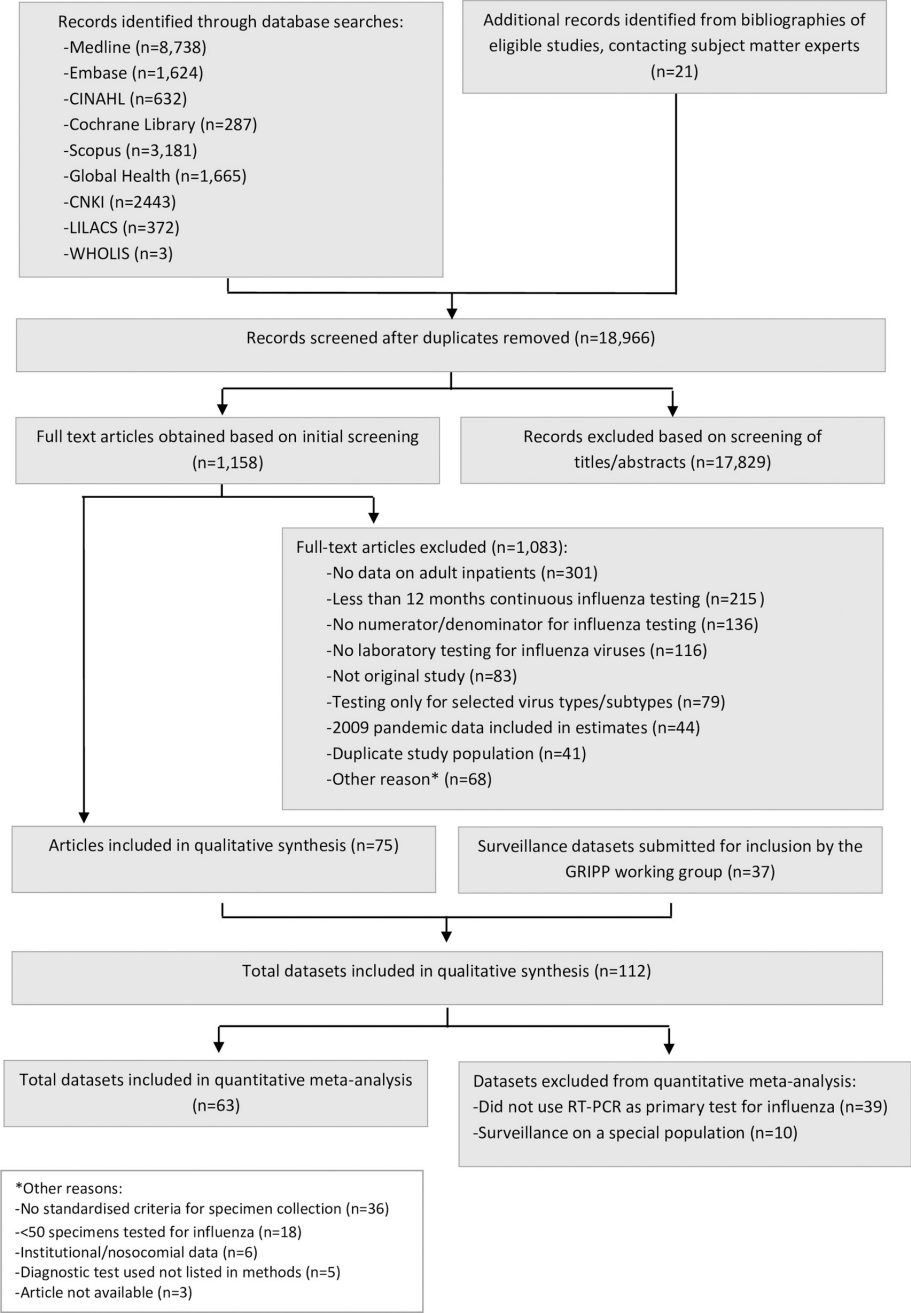
Flow diagram for the systematic review process. GRIPP, Global Respiratory Hospitalizations–Influenza Proportion Positive.

The systematic literature search identified 18,945 unique articles published between January 1996 and December 2016, describing data collected from 1982 through 2016. An additional 21 articles were identified outside the search through consultation with subject-matter experts and review of references. Of these 18,966 articles, we reviewed 1,158 full-text articles; 75 met the inclusion criteria and were abstracted for the descriptive analysis (Fig 1). Combining these with the 37 GRIPP Working Group surveillance datasets, we included 112 unique influenza data sources from 55 countries across all 6 WHO regions. Compared with published articles, surveillance datasets were more likely to use RT-PCR as the primary method of diagnosis (*n =* 35/37, 95%, versus *n =* 38/75, 51%; *p <* 0.001) and more likely to use SARI, a WHO-recommended case definition for severe influenza disease [19], as the case definition (78% versus 8%, *p <* 0.001). Surveillance datasets were also more commonly from low- and lower-middle income countries, compared with published articles (67% versus 14%, *p <* 0.001). Approximately 57% of published articles presented <2 years of data, versus 8% of surveillance datasets (*p <* 0.001). Special populations were the specific focus of 27% (*n =* 20/75) of the published articles, and none of the surveillance datasets (Table 1). Frequency of comorbid conditions was reported for 47 (63%) of the 75 published articles, with a range of 25% to 100% of patients having 1 or more conditions. Influenza vaccination status of patients was only reported by 5 articles; vaccine coverage ranged between 7% and 48%.

**Table 1:** Description of published studies and Global Respiratory Hospitalizations–Influenza Proportion Positive (GRIPP) surveillance datasets summarizing influenza-associated respiratory hospitalizations among adults, 1996–2016.

Characteristic	Published studies, *n =* 75		Surveillance datasets, *n =* 37		*p*-Value
	*n*	Percent	*n*	Percent	
**Age group**					
Adults <65 years*	14	19%	36	97%	<0.001
Adults ≥65 years	32	43%	16	43%	0.95
All adults	75	100%	37	100%	
**Study duration in years**					<0.001
1 to 2	43	57%	3	8%	
3 to 4	31	41%	14	38%	
5+	1	1%	20	54%	
**Timeframe**					<0.001
Pre-2009 data only	45	60%	0	0%	
Post-2009 data	30	40%	31	84%	
Both pre- and post-2009 data	0	0%	6	16%	
**Total number of specimens tested**					<0.001
50–99	12	16%	1	3%	
100–499	48	64%	7	19%	
500–999	9	12%	5	14%	
1,000+	6	8%	24	65%	
**Diagnostic test**					<0.001
Reverse transcription PCR (RT-PCR) only	23	31%	17	46%	
Immunofluorescence only	12	16%	0	0%	
Serological assay only	13	17%	0	0%	
Multiple tests, including RT-PCR	18	24%	20	54%	
Multiple tests, excluding RT-PCR	6	8%	0	0%	
Other^†^	3	4%	0	0%	
**Case definition**					<0.001
Acute respiratory infection (ARI)	6	8%	1	3%	
Lower respiratory infection (LRI)	3	4%	5	14%	
Pneumonia	32	43%	0	0%	
Severe acute respiratory infection (SARI)	6	8%	29	78%	
Other^‡^	28	37%	2	5%	
**Special population**^¶^					<0.001
Yes	20	27%	0	0%	
No	55	73%	37	100%	
**World Health Organization region**					<0.001
Africa	1	1%	8	22%	
Americas	11	15%	7	19%	
Eastern Mediterranean	4	5%	4	11%	
Europe	22	29%	1	3%	
Southeast Asia	5	7%	9	24%	
Western Pacific	32	43%	8	22%	
**World Bank income level**					<0.001
Low	1	1%	9	24%	
Lower-middle	10	13%	16	43%	
Upper-middle	24	32%	7	19%	
High	40	53%	5	14%	
**Quality score (published studies only)**^**§**^					
0	2	3%			
1	17	23%			
2	36	48%			
3	20	27%			
**Included in meta-analysis**					<0.001
Yes	28	37%	35	95%	
No	47	63%	2	5%	

The range of study years for the published studies was 1982–2016; the range of study years for the surveillance datasets was 2005–2016.

*Gaps in age-specific estimates due to lack of outcome data stratified by age group.

^†^Includes rapid immunochromatographic assay (*n =* 2) and virus culture (*n =* 1).

^‡^Includes acute exacerbation of chronic obstructive pulmonary disease (COPD) (*n =* 14), respiratory hospitalization (acute or non-acute) (*n =* 10), acute exacerbation of asthma (*n =* 3), acute febrile illness (*n =* 2), intubation among intensive care unit (ICU) patients (*n =* 1), acute respiratory distress syndrome (ARDS) (*n =* 1), ICD codes consistent with influenza (*n =* 1), and acute coronary syndrome with recent influenza-like illness (*n =* 1).

^¶^Includes studies of populations with COPD (*n =* 10), hospitalized in the ICU only (*n =* 3), with immunocompromising conditions (*n =* 1), with asthma (*n =* 1), with heart disease (*n =* 1), or in the military (*n =* 1).

^§^Quality of included articles was assessed using a simplified Newcastle–Ottawa scale [9,15]. Possible quality score from 0 (lowest) to 3 (highest).

Among all data sources (*n =* 112), the median percent of respiratory samples that were influenza positive among adult patients <65 years was similar to that among those ≥65 years (15%, IQR 9%–21%, versus 13%, IQR 8%–17%, respectively; *p =* 0.280). The median percent positive was higher among surveillance data compared to published articles (15%, IQR 10%–20%, versus 10%, IQR 6%–17%; *p =* 0.011). The median percent positive was higher among middle-income countries than low- or high-income countries (*p =* 0.035). Case definition was also influential, with higher values among studies using SARI or LRI compared to those using acute respiratory infection (ARI) or pneumonia. The percent of patients positive for influenza was not associated with diagnostic test used (S3 Table). When we restricted the data to the subset of studies that were included in the meta-analysis—i.e., those using RT-PCR data (excluding special populations), shown in Table 2—the median proportion positive differed only by case definition, as indicated by post hoc non-parametric pairwise testing (Dunn’s test with Bonferroni correction), which identified a significant difference between pneumonia-based estimates and SARI-based estimates (*p =* 0.005).

**Table 2:** Median number of specimens tested and percent positive for influenza, by age group, study design, and population, among studies included in the meta-analysis.

Category	Number of data sources (*n =* 63)	Total number of specimens tested			Percent influenza-positive			*p-*Value
		Median	IQR		Median	IQR		
			Lower bound	Upper bound		Lower bound	Upper bound	
**All adults**	63	510	225	2,092	13%	8%	19%	
Age group*								
18–64 years	41	867	341	2,759	16%	11%	21%	0.17
65+ years	38	198	81	890	14%	9%	17%	
**Data source**								
Surveillance	35	1,376	542	3,775	15%	10%	20%	0.089
Published	28	225	120	349	10%	7%	18%	
**Timeframe**								
Pre-2009 data	14	191	124	242	9%	8%	12%	0.097
Post-2009 data	45	987	328	2,818	15%	9%	20%	
Pre- and post-2009 data	4	5,948	2,581	9,527	10%	10%	13%	
**Case definition**								
Acute respiratory infection (ARI)	6	254	126	279	10%	4%	13%	0.006
Lower respiratory infection (LRI)	7	1,158	513	6,140	13%	11%	15%	
Pneumonia	12	182	120	258	8%	6%	10%	
Severe acute respiratory infection (SARI)	33	1,720	426	3,222	17%	11%	21%	
Other^†^	5	510	256	582	10%	9%	17%	
**World Health Organization region**								
Africa	8	1,490	491	1,797	9%	6%	11%	0.071
Americas	11	328	210	4,081	13%	7%	18%	
Eastern Mediterranean	7	987	361	2,743	20%	20%	22%	
Europe	8	191	134	262	9%	7%	11%	
Southeast Asia	11	1,130	456	2,573	14%	11%	18%	
Western Pacific	18	485	259	2,458	15%	10%	19%	
**World Bank income level**								
Low	9	1,253	565	1,766	12%	11%	14%	0.14
Lower-middle	20	785	312	2,952	16%	10%	21%	
Upper-middle	16	467	237	1,737	15%	9%	21%	
High	18	294	188	1,822	10%	7%	15%	

*Gaps in age-specific estimates due to lack of outcome data stratified by age group.

^†^Includes respiratory hospitalization (acute or non-acute) (*n =* 4) and ICD codes consistent with influenza (*n =* 1).

**Fig 2:**
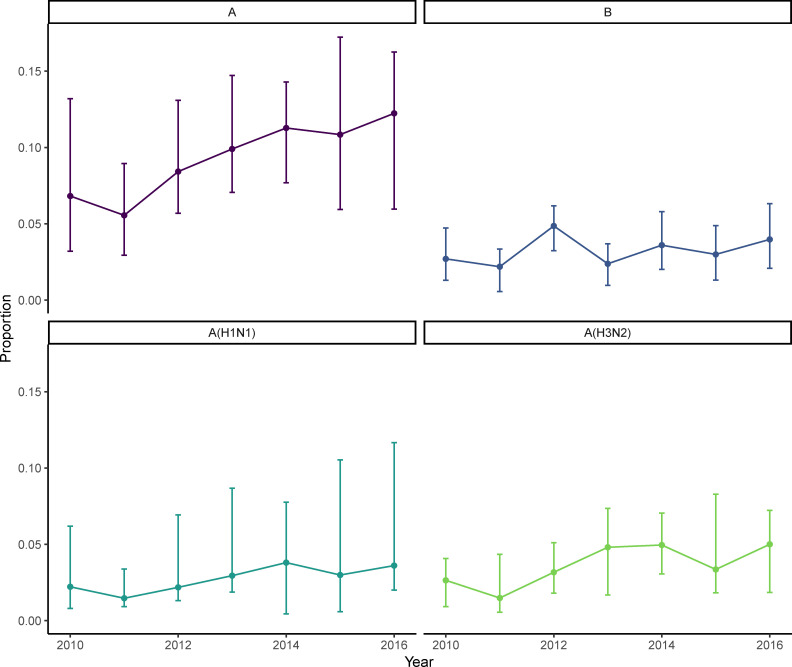
Median proportion of adult respiratory samples testing positive for influenza virus by year and virus type/subtype, 2010 to 2016. Error bars indicate interquartile range for each estimate.

Data by year and influenza A virus subtype were primarily available from surveillance conducted by the GRIPP Working Group in years after the 2009 pandemic (Fig 2). While different virus types and subtypes predominated from year to year within individual datasets, the yearly median percent positive across datasets showed that influenza A viruses were detected approximately 3 times more frequently than influenza B viruses, with generally equal frequency of influenza A(H1N1) and influenza A(H3N2) viruses in any given year. An increasing tendency was seen in influenza A (overall and both subtypes), but not influenza B, during this time.

The primary meta-analysis model (*n =* 63 datasets) found influenza to be associated with 14.1% (95% CI 12.1%–16.5%) of acute respiratory hospitalizations (all adults). Influenza A viruses were associated with an estimated 10.6% (95% CI 8.9%–12.5%) of these episodes, and influenza B viruses with 3.5% (95% CI 2.8%–4.3%). Applying the overall percent influenza positive to estimates of hospitalized and total LRI episodes resulted in a total of 5,678,000 (95% CI 3,205,000–9,432,000) influenza-associated LRI hospitalizations and 32,126,000 (95% CI 20,484,000–46,129,000) influenza-associated LRI episodes in persons ≥20 years globally. Using age-specific proportion positive estimates, this equated to 3,464,000 (95% CI 1,885,000–5,978,000) influenza-associated hospitalizations among adults 20–64 years and 2,831,000 (95% CI 1,716,000–3,969,000) influenza-associated hospitalizations among adults ≥65 years (Table 3). From these estimates, we calculated a rate of 80 (95% CI 44–139) hospitalizations/100,000 population among adults <65 years and 437 (95% CI 265–612) hospitalizations/100,000 population among older adults. By virus type, we estimated 4,264,000 (95% CI 2,185,000–7,353,000) influenza A–associated and 1,408,000 (95% CI 322,000–3,034,000) influenza B–associated hospitalizations each year, as well as 24,126,000 (95% CI 13,880,000–36,677,000) influenza A–associated and 7,966,000 (95% CI 1,650,000–15,426,000) influenza B–associated LRI episodes. Regional estimates are presented in S4 Table. Additional model data and sensitivity analyses are presented in S5 Table. A forest plot of findings by dataset is presented in S1 Fig.

**Table 3:** Global estimates of influenza-associated lower respiratory infection (LRI) episodes and hospitalizations, by age group.

Estimated outcome	All-cause estimate* (thousands) (95% CI)	Pooled percent influenza-positive^†^ (95% CI)	Number of datasets	Influenza-associated events (thousands) (95% CI)	Rate per 100,000 population (95% CI)
**All adults—any influenza**					
Hospitalized LRI episodes	40,226 (25,810–59,569)	14.1% (12.1%–16.5%)	63	5,678 (3,205–9,432)	115 (65–190)
LRI episodes	227,603 (190,403–268,571)			32,126 (20,484–46,129)	649 (414–931)
**All adults—influenza A**					
Hospitalized LRI episodes	40,226 (25,810–59,569)	10.6% (8.9%–12.5%)	51	4,264 (2,185–7,353)	86 (44–148)
LRI episodes	227,603 (190,403–268,571)			24,126 (13,880–36,677)	487 (280–740)
**All adults—influenza B**					
Hospitalized LRI episodes	40,226 (25,810–59,569)	3.5% (2.8%–4.3%)	46	1,408 (322–3,034)	28 (7–61)
LRI episodes	227,603 (190,403–268,571)			7,966 (1,650–15,426)	161 (33–311)
**Adults 20–64 years—any influenza**					
Hospitalized LRI episodes	20,939 (12,894–32,704)	16.5% (13.7%–19.8%)	41	3,464 (1,885–5,978)	80 (44–139)
LRI episodes	187,893 (155,794–223,148)			31,087 (19,987–44,444)	722 (464–1,032)
**Adults ≥65 years—any influenza**					
Hospitalized LRI episodes	19,287 (12,916–26,866)	14.7% (12.7%–16.8%)	38	2,831 (1,716–3,969)	437 (265–612)
LRI episodes	39,709 (34,609–45,423)			5,830 (4,927–6,740)	899 (760–1,040)

*All-cause estimates are derived from the Global Burden of Disease project, adapted for 2016, among individuals 20 years and older.

^†^Percent influenza-positive and number of datasets from meta-analysis.

## Discussion

We estimated that seasonal influenza viruses were associated with 14% of acute lower respiratory disease among hospitalized adults. In 2016, this equated to 5.7 million influenza-associated LRI hospitalizations and 32.1 million LRI episodes globally. The majority of hospitalizations and episodes occurred among younger adults, although influenza-associated hospitalization rates were 5 times higher among older adults. Three-quarters of episodes (24 million) were associated with influenza A viruses of any subtype, and one-quarter (8 million) were associated with influenza B viruses.

These estimates are consistent with, or slightly higher than, existing global estimates of influenza morbidity and mortality. Our model adds to the existing knowledge by providing an estimate focused specifically on adult populations, adding a large amount of additional influenza-specific data from unpublished surveillance data over several years from a diverse range of countries where publications are often not available, and allowing for validation and triangulation across models. The 2017 GBD study found that influenza was associated with 9.5 million LRI hospitalizations worldwide among all age groups, with a majority of these occurring among children 19 years and younger. This estimate was based on an attributable fraction estimate for all ages of 8.5%, derived from a systematic literature review of published studies, but was lower than our estimate for adults (14%), as well as our previous estimate for older children (16%) [8,10]. The GBD study’s approach might underestimate the attributable fraction of influenza among adults because of the inclusion of pediatric patients, who are less likely to test positive for influenza. The attributable fraction used in the GBD analysis also included data derived from less-sensitive diagnostic assays (i.e., ELISA) in addition to RT-PCR–diagnosed influenza [20].

By comparing our estimated LRI episodes and hospitalizations to published estimates of global influenza mortality, we are also able to validate our estimated outcomes. Recent estimates suggest that influenza is associated with 291,243–645,832 deaths per year [9]. Assuming the majority of these deaths occur among adults, and using the upper bound of this estimate, this would translate to 11% of influenza-associated hospitalizations and 2% of influenza-associated LRI episodes, which are consistent with US Centers for Disease Control and Prevention estimates of the burden of disease for influenza [21].

This analysis is subject to several limitations. Our model summarizes the magnitude of influenza-associated hospitalizations across many years to determine the proportion that are influenza-associated in any 1 year. However, the proportion of respiratory disease caused by influenza in a given setting or year can be affected by a wide range of factors, such as specimen type, case definition, influenza and pneumococcal vaccine use, characteristics of predominating influenza viruses, and trends in circulation of other pathogens. We attempted to understand and control for these factors in the current analysis through our inclusion criteria (sampling process used, year-round diagnostic testing) and stratified analysis (by case definition and other factors). The proportion of patients with respiratory disease who are hospitalized is also affected by many factors, such as access to care and health-seeking behavior; diagnostic, admission, and referral practices; age; and underlying health status, which likely differ by study site. An additional challenge for this analysis was the relatively limited amount of or lack of stratified data (by year, age group, virus subtype, presence of underlying conditions, region, vaccination history) from both published and unpublished data sources to make for robust stratified analyses (e.g., by region). Further, laboratory diagnosis of influenza in a case of adult LRI may not mean that influenza infection was the cause of hospitalization, as we did not account for other pathogens or underlying factors. Finally, our estimates for LRI episodes are generated with a proportion influenza positive derived from hospitalized illnesses. Total LRI episodes include illnesses that may or may not be hospitalized. In lower-income settings access to hospital services can be limited; therefore, individuals with illnesses who do not seek care are likely to share similar characteristics with those who do. The proportion associated with influenza may be similar between those who are and are not hospitalized in these settings. Moreover, previous global analyses [10] indicate that non-severe LRI is often more likely to be influenza-positive than severe LRI, and thus applying the hospitalized percent positive to potentially milder non-hospitalized LRI cases suggests that we may have underestimated the true burden of total influenza-associated LRI episodes worldwide.

### Conclusions

The burden of severe influenza-associated disease and hospitalizations among adults worldwide is substantial. While older adults, young children, pregnant women, and those with chronic illness are already recommended as target groups for vaccination by WHO, influenza vaccine uptake is low globally, and the widely-used standard-dose inactivated influenza vaccines may have a lower effectiveness in key populations such as older adults, compared to younger adult populations [22]. Efforts to improve influenza vaccines (e.g., via high-dose or adjuvanted formulations) and expand vaccination programs in low- and middle-income countries could lead to more routine vaccination and thus increase protection against severe influenza disease [23–25]; however, these initiatives require substantial investment of time and resources.

Given these challenges, and variations in incidence within and between nations and regions, high-quality surveillance data will continue to play a pivotal role in informing influenza prevention and control measures, particularly in elucidating influenza virus infection’s role as a cause of severe respiratory disease and hospitalizations among adults. Moreover, with the emergence of SARS-CoV-2 this study emphasizes the critical importance of unbiased surveillance using standard case definitions in a wide range of settings in understanding the global and regional impact of respiratory diseases.

## Supporting information

S1 Data CSV-formatted analysis dataset. (CSV)

S1 Fig Forest plot of individual study estimates included in meta-analysis (all adults). (TIF)

S1 PRISMA Checklist PRISMA checklist. (PDF)

S1 Table Literature search methodology and results, by database. (PDF)

S2 Table Summary of published articles included in the analyses, with reference list. (PDF)

S3 Table Median number of specimens tested and percent positive for influenza, by age group, study design, and population, among all data sources. (PDF)

S4 Table Regional estimates of influenza-associated lower respiratory infection (LRI) episodes and hospitalizations, by age group. (PDF)

S5 Table Sensitivity analyses. (PDF)

## Abbreviations

GBD: Global Burden of Disease
GRIPP: Global Respiratory Hospitalizations–Influenza Proportion Positive
LRI: lower respiratory infection
RT-PCR: reverse transcription PCR
SAGE: Strategic Advisory Group of Experts on Immunization
SARI: severe acute respiratory infection

## References

[1] Thompson WW, Shay DK, Weintraub E, Brammer L, Cox N, Anderson LJ, et al. Mortality associated with influenza and respiratory syncytial virus in the United States. JAMA. 2003;289(2):179–86. 10.1001/jama.289.2.179 12517228

[2] Feng L, Shay DK, Jiang Y, Zhou H, Chen X, Zheng Y, et al. Influenza-associated mortality in temperate and subtropical Chinese cities, 2003–2008. Bull World Health Organ. 2012;90(4):279–88B. 10.2471/BLT.11.096958 22511824 PMC3324869

[3] Poehling KA, Edwards KM, Griffin MR, Szilagyi PG, Staat MA, Iwane MK, et al. The burden of influenza in young children, 2004–2009. Pediatrics. 2013;131(2):207–16. 10.1542/peds.2012-1255 23296444 PMC3557405

[4] Yokomichi H, Mochizuki M, Lee JJ, Kojima R, Yokoyama T, Yamagata Z. Incidence of hospitalisation for severe complications of influenza virus infection in Japanese patients between 2012 and 2016: a cross-sectional study using routinely collected administrative data. BMJ Open. 2019;9(1):e024687. 10.1136/bmjopen-2018-024687 30782739 PMC6340484

[5] Chaves SS, Aragon D, Bennett N, Cooper T, D’Mello T, Farley M, et al. Patients hospitalized with laboratory-confirmed influenza during the 2010–2011 influenza season: exploring disease severity by virus type and subtype. J Infect Dis. 2013;208(8):1305–14. 10.1093/infdis/jit316 23863950

[6] Meeting of the Strategic Advisory Group of Experts on immunization, April 2012—conclusions and recommendations. Wkly Epidemiol Rec. 2012;87(21):201–16. 24340402

[7] World Health Organization. WHO SAGE Seasonal Influenza Vaccination Recommendations during the COVID-19 pandemic: interim guidance. Geneva: World Health Organization; 2020 [cited 2021 Feb 25]. Available from: https://www.who.int/immunization/policy/position_papers/Interim_SAGE_influenza_vaccination_recommendations.pdf?ua=1.

[8] Lafond KE, Nair H, Rasooly MH, Valente F, Booy R, Rahman M, et al. Global role and burden of influenza in pediatric respiratory hospitalizations, 1982–2012: a systematic analysis. PLoS Med. 2016;13(3):e1001977. 10.1371/journal.pmed.1001977 27011229 PMC4807087

[9] Iuliano AD, Roguski KM, Chang HH, Muscatello DJ, Palekar R, Tempia S, et al. Estimates of global seasonal influenza-associated respiratory mortality: a modelling study. Lancet. 2018;391(10127):1285–300. 10.1016/S0140-6736(17)33293-2 29248255 PMC5935243

[10] GBD 2017 Influenza Collaborators. Mortality, morbidity, and hospitalisations due to influenza lower respiratory tract infections, 2017: an analysis for the Global Burden of Disease Study 2017. Lancet Respir Med. 2019;7(1):69–89. 10.1016/S2213-2600(18)30496-X 30553848 PMC6302221

[11] Bresee J, Fitzner J, Campbell H, Cohen C, Cozza V, Jara J, et al. Progress and remaining gaps in estimating the global disease burden of influenza. Emerg Infect Dis. 2018;24(7):1173–7. 10.3201/eid2407.171270 29912681 PMC6038739

[12] World Bank. World Bank GNI per capita operational guidelines & analytical classifications: country analytical history. Washington (DC): World Bank; 2020 [cited 2021 Feb 25]. Available from: http://databank.worldbank.org/data/download/site-content/OGHIST.xls.

[13] World Health Organization. WHO-UNICEF estimates of PCV3 coverage. Geneva: World Health Organization; 2019 [cited 2021 Feb 25]. Available from: http://apps.who.int/immunization_monitoring/globalsummary/timeseries/tswucoveragepcv3.html.

[14] Wells GA, Shea B, O’Connell D, Peterson J, Welch V, Losos M, et al. The Newcastle-Ottawa Scale (NOS) for assessing the quality of nonrandomised studies in meta-analyses. Ottawa: Ottawa Hospital Research Institute; 2019 [cited 2019 Dec 6]. Available from: http://www.ohri.ca/programs/clinical_epidemiology/oxford.asp.

[15] Moher D, Liberati A, Tetzlaff J, Altman DG. Preferred reporting items for systematic reviews and meta-analyses: the PRISMA statement. PLoS Med. 2009;6(7):e1000097. 10.1371/journal.pmed.1000097 19621072 PMC2707599

[16] United Nations Population Division. World population prospects 2019. New York: United Nations Population Division; 2021 [cited 2021 Feb 25]. Available from: https://population.un.org/wpp/.

[17] R Core Team. R: a language and environment for statistical computing. Vienna: R Foundation for Statistical Computing; 2018 [cited 2021 Feb 25]. Available from: https://www.R-project.org/.

[18] Bates D, Maechler M, Bolker B, Walker S. Fitting linear mixed-effects models using lme4. J Stat Softw. 2015;67(1):1–48.

[19] World Health Organization. WHO surveillance case definitions for ILI and SARI. Geneva: World Health Organization; 2014 [cited 2021 Feb 25]. Available from: https://www.who.int/influenza/surveillance_monitoring/ili_sari_surveillance_case_definition/en/.

[20] Vemula SV, Zhao J, Liu J, Wang X, Biswas S, Hewlett I. Current approaches for diagnosis of influenza virus infections in humans. Viruses. 2016;8(4):96. 10.3390/v8040096 27077877 PMC4848591

[21] US Centers for Disease Control and Prevention. Disease burden of influenza. Atlanta: US Centers for Disease Control and Prevention; 2020 [cited 2021 Feb 25]. Available from: https://www.cdc.gov/flu/about/burden/index.html.

[22] Osterholm MT, Kelley NS, Sommer A, Belongia EA. Efficacy and effectiveness of influenza vaccines: a systematic review and meta-analysis. Lancet Infect Dis. 2012;12(1):36–44. 10.1016/S1473-3099(11)70295-X 22032844

[23] Gravenstein S, Davidson HE, Taljaard M, Ogarek J, Gozalo P, Han L, et al. Comparative effectiveness of high-dose versus standard-dose influenza vaccination on numbers of US nursing home residents admitted to hospital: a cluster-randomised trial. Lancet Respir Med. 2017;5(9):738–46. 10.1016/S2213-2600(17)30235-7 28736045

[24] Domnich A, Arata L, Amicizia D, Puig-Barbera J, Gasparini R, Panatto D. Effectiveness of MF59-adjuvanted seasonal influenza vaccine in the elderly: a systematic review and meta-analysis. Vaccine. 2017;35(4):513–20. 10.1016/j.vaccine.2016.12.011 28024956

[25] Bresee JS, Lafond KE, McCarron M, Azziz-Baumgartner E, Chu SY, Ebama M, et al. The partnership for influenza vaccine introduction (PIVI): supporting influenza vaccine program development in low and middle-income countries through public-private partnerships. Vaccine. 2019;37(35):5089–95. 10.1016/j.vaccine.2019.06.049 31288998 PMC6685526

