# Supplementary figures and images for: Global burden of influenza-associated lower respiratory tract infections and hospitalizations among adults: A systematic review and meta-analysis
Source: PLoS Med. 2021 Mar 1;18(3):e1003550. doi: 10.1371/journal.pmed.1003550 (PMC7959367; doi:10.1371/journal.pmed.1003550)

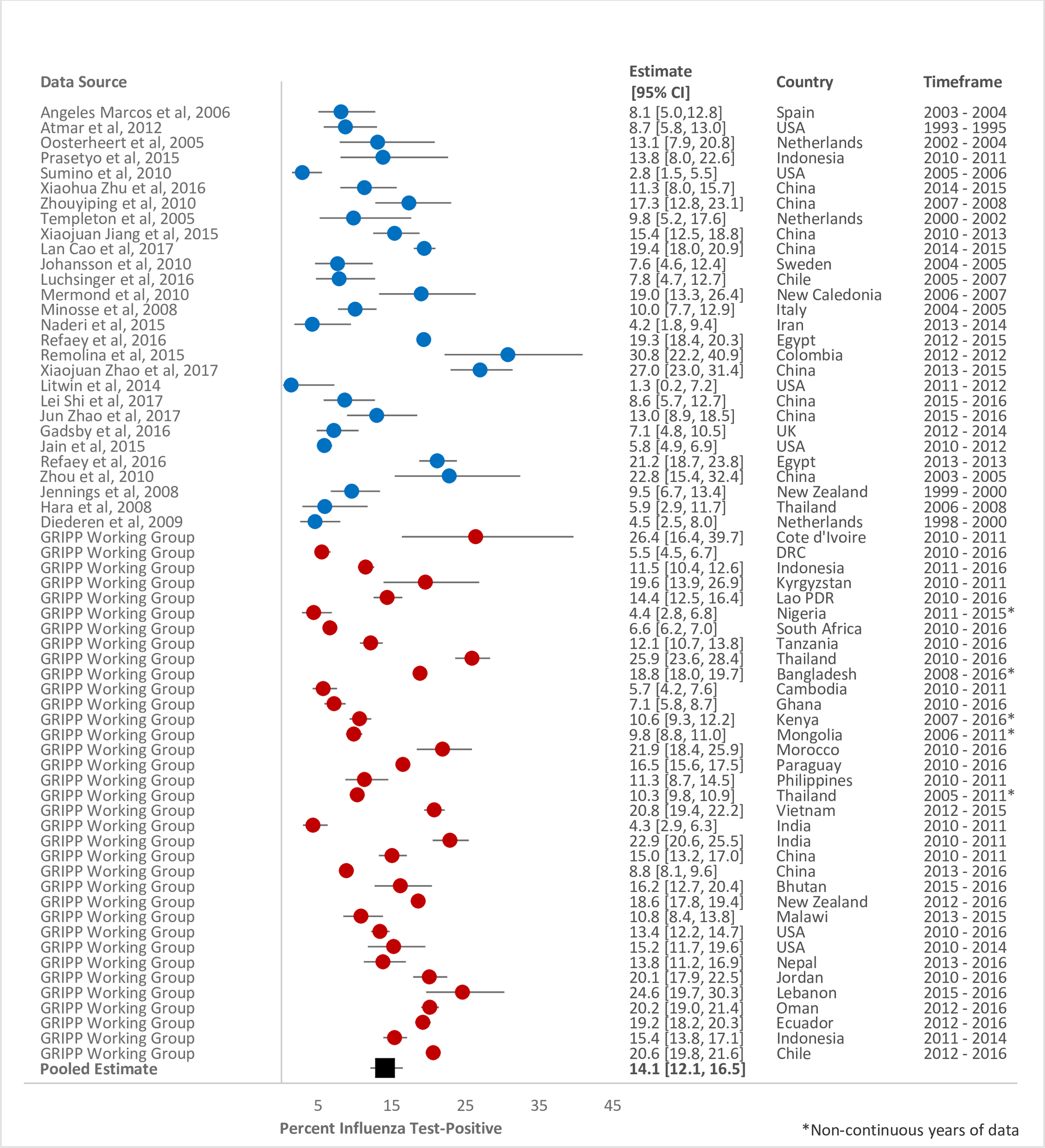

Supplement: S1 Fig — (TIF) [file pmed.1003550.s002.tif]
