# Supplementary material for: Global burden of influenza-associated lower respiratory tract infections and hospitalizations among adults: A systematic review and meta-analysis
Source: PLoS Med. 2021 Mar 1;18(3):e1003550. doi: 10.1371/journal.pmed.1003550 (PMC7959367; doi:10.1371/journal.pmed.1003550)
Supplement: S1 Table — (PDF) [file pmed.1003550.s004.pdf]

Supplementary Table 1. Literature search methodology and results, by database

Database	Search Terminology	Run date	Results	Number kept[1]
**Medline (OVID) 1946–**	(Influenza OR flu).mp<br>AND<br>(hospital* OR inpatient* OR in-patient* OR health* facility* OR indoor patient* OR admitted OR admission* OR intensive care OR acute care OR ICU).mp	1/26/17	8755	8738
**Embase (OVID) 1996–**	(Influenza OR flu)<br>AND<br>(Hospital* OR inpatient* OR in-patient* OR health* facilit* OR indoor patient* OR admitted OR admission* OR intensive care OR acute care OR ICU)<br>Exclude Medline Journals	1/26/2017	1858	1624
**CINAHL (Ebsco) 1982–**	(Influenza OR flu)<br>AND<br>(Hospital* OR inpatient* OR in-patient* OR health* facilit* OR indoor patient* OR admitted OR admission* OR intensive care OR acute care OR ICU)<br>Exclude Medline Records	1/27/2017	726	632
**Cochrane Library**	(Influenza OR flu):ti,ab<br>AND<br>(Hospital* OR inpatient* OR in-patient* OR health* facilit* OR indoor patient* OR admitted OR admission* OR intensive care OR acute care OR ICU):ti,ab	1/27/2017	563	287
**Scopus**	TITLE-ABS-KEY((Influenza OR flu) AND (Hospital* OR inpatient* OR in-patient* OR (health W/2 facilit*) OR "indoor patient*" OR admitted OR admission* OR "intensive care" OR "acute care" OR ICU)) AND NOT INDEX(medline)	1/27/2017	5669	3181
**Global Health (OVID) 1973–**	(Influenza OR flu)<br>AND<br>(Hospital* OR inpatient* OR in-patient* OR health* facilit* OR indoor patient* OR admitted OR admission* OR intensive care OR acute care OR ICU)	1/26/2017	5870	1665
**LILACS IBECS**	(tw:(influenza OR flu)) AND (tw:(hospital OR hospitals OR hospitalized OR inpatient OR inpatients OR indoor patient OR indoor patients))	1/27/2017	419	372
**WHOLIS**	(tw:(influenza OR flu)) AND (tw:(hospital OR hospitals OR hospitalized OR inpatient OR inpatients OR indoor patient OR indoor patients))	1/27/2017	3	3
**China Knowledge Resource Integrated Database (CNKI) 1979–**	SU=流感 AND (SU=医院 OR SU=住院病人 OR SU=卫生设施 OR SU=住院 OR SU=入院 OR SU=重症监护 OR SU=危重病人医疗 OR SU=重症监护病房)	10/27/2017	2456	2443

[1]Duplicates were identified and removed using the Endnote automated “find duplicates” function with preference set to match on title, author, and year.
