# Supplementary material for: Global burden of influenza-associated lower respiratory tract infections and hospitalizations among adults: A systematic review and meta-analysis
Source: PLoS Med. 2021 Mar 1;18(3):e1003550. doi: 10.1371/journal.pmed.1003550 (PMC7959367; doi:10.1371/journal.pmed.1003550)
Supplement: S2 Table — (PDF) [file pmed.1003550.s005.pdf]

Supplementary Table 2. Summary of published articles included in the analyses, with reference list

Location	Years	Case definition	Age range	% Influenza positive, all ages	% Influenza positive, 18–64 years	% Influenza positive, ≥65 years	PCR testing	Quality Score [1]
Kuopia, Finland [2]	1981–1982	Pneumonia	-	2.2	6.7	0.0	No	3
Beer-Sheva, Israel [3]	1991–1992	CAP	≥17	5.8	-	-	No	2
Multiple sites, Spain [4]	1992–1994	CAP among COPD patients*	-	0.0	-	-	No	2
Melbourne, Australia [5]	1993–1994	AE-asthma*	17–66	25.3	-	-	No	1
Houston, USA [6]	1993–1995	Acute respiratory condition	≥18	8.7	9.5	6.5	Yes	2
Coast Province, Kenya [7]	1994–1996	Acute pneumonia	≥15	6.1	-	-	No	2
Okayama, Japan [8]	1994–1997	CAP	>15	1.4	-	-	No	2
Singapore, Singapore [9]	1995–1997	AE-COPD*	-	24.4	-	-	No	2
Ljubljana, Slovenia [10]	1996–1997	CAP	>15	1.9	-	-	No	3
Barcelona, Spain [11]	1996–2001	CAP	≥18	11.5	-	-	No	3
Beer-Sheva, Israel [12]	1997–1999	AE-COPD*	>40	15.8	-	-	No	2
Georgia, USA [13]	1997–1999	Febrile respiratory illness in military population*	≥17	8.5	8.5	-	No	2
Dalian, China [14]	1997–2000	CAP	15–91	16.5	18.0	14.5	No	3
Nottingham, UK [15]	1998–1999	CAP	≥16	20.8	-	19.6	No	3
Bochum, Germany [16]	1998–1999	AE-COPD*	≥18	22.4	-	-	Yes	3
Schiedam, Netherlands [17]	1998–1999	CAP	≥20	21.7	-	-	No	2
Alkmaar, Netherlands [18]	1998–2000	CAP	≥18	4.5	-	-	Yes	2
Buenos Aires, Argentina [19]	1998–2001	CAP	≥16	20.0	-	-	No	2
Nakasange, Japan [20]	1998–2000	CAP	≥65	7.1	-	7.1	No	2
Christchurch, New Zealand [21]	1999–2000	CAP	≥18	9.5	-	-	Yes	3
Turku, Finland [22]	1999–2004	CAP	≥16	7.8	-	-	No	2
Al-Adan, Kuwait [23]	2000–2001	CAP	≥15	10.5	-	-	No	2
Leiden, Netherlands [24]	2000–2002	CAP	≥18	9.8	-	-	Yes	1
Newcastle, Australia [25]	2000–2003	AE-COPD*	>45	17.8	-	-	Yes	3
Multiple Sites, Thailand [26]	2001–2002	Acute febrile illness	15-87	11.1	-	-	No	1
Utrecht, Netherlands [27]	2002–2004	LRTI	≥18	13.1	-	-	Yes	2
Santiago, Chile [28]	2003–2004	CAP	>14	6.9	-	-	No	3
Barcelona, Spain [29]	2003–2004	CAP	>14	8.1	-	-	Yes	2
Caen, France [30]	2003–2004	Intubation in ICU patients*	-	3.7	-	-	Yes	3
Cologne, Germany [31]	2003–2004	Atypical pneumonia in immunocompromised patients*	≥18	1.6	-	-	No	1
Melbourne, Australia [32]	2003–2005	AE-COPD*	≥49	2.0	-	-	Yes	3

Location	Years	Case definition	Age range	% Influenza positive, all ages	% Influenza positive, 18–64 years	% Influenza positive, ≥65 years	PCR testing	Quality Score [1]
Shenzhen, China [33]	2003–2005	Pneumonia	18–84	22.8	-	-	Yes	2
Stockholm, Sweden [34]	2004–2005	CAP	≥18	7.6	-	-	Yes	3
Hong Kong, Hong Kong [35]	2004–2005	AE-COPD with pneumonia*	-	6.7	-	-	Yes	0
Rome, Italy [36]	2004–2005	Pulmonary disease	-	10.0	-	-	Yes	2
Edmonton, Canada [37]	2004–2006	CAP	-	3.6	-	-	No	3
Puerto Montt, Chile [38]	2005–2006	Pneumonia	>15	6.3	-	-	No	1
St- Louis, USA [39]	2005–2006	Acute respiratory illness	-	2.8	-	-	Yes	2
Santiago, Chile [40]	2005–2007	CAP	≥18	7.8	-	-	Yes	1
Noumea, New Caledonia [41]	2006–2007	CAP	≥16	19.0	-	-	Yes	3
Chiang Mai, Thailand [42]	2006–2007	ACS with recent ILI*	-	12.5	-	-	No	0
Chiang Mai, Thailand [43]	2006–2008	CAP	≥16	5.9	-	-	Yes	2
Geneva, Switzerland [44]	2007–2008	AE-COPD*	-	2.3	-	-	Yes	2
Shenzhen, China [45]	2007–2008	Respiratory infection	16–89	17.3	18.2	15.8	Yes	1
Turin, Italy [46]	2008–2008	Fever and/or respiratory symptoms	≥16	0.6	-	-	No	3
Seoul, Korea [47]	2010–2011	AE-COPD*	-	3.2	-	-	Yes	1
Surakarta, Indonesia [48]	2010–2011	ILI, ARI, or SARI	-	13.8	-	-	Yes	1
Chicago/Nashville, USA [49]	2010–2012	CAP	≥18	5.8	-	-	Yes	2
Seoul, Korea [50]	2010–2012	AE-COPD, including pneumonia*	-	10.3	-	-	Yes	2
Kashmir, India [51]	2010–2012	AE-COPD*	≥40	8.0	-	-	Yes	3
Gansu, China [52]	2010–2013	LRTI	≥15	15.4	-	9.0	Yes	1
Haidlan/Beijing, China [53]	2010–2013	AE-asthma*	18–63	8.9	8.9	-	No	2
Georgia, USA [54]	2011–2012	Respiratory infection	≥22	1.3	-	-	Yes	1
Florence, Italy [55]	2011–2014	ARDS*	-	35.2	-	-	Yes	1
Huangshi/Hubei, China [56]	2011–2014	Respiratory illness	50–80	3.4	0.0	5.6	No	1
Bogota, Colombia [57]	2012–2012	SARI	≥18	30.8	-	-	Yes	3
Edinburgh, UK [58]	2012–2014	CAP	-	7.1	-	-	Yes	2
Multiple Sites, Egypt [59]	2012–2015	SARI	≥15	19.3	20.0	15.5	Yes	2
Damanhour, Egypt [60]	2013–2013	SARI	≥15	21.2	22.3	13.3	Yes	3
Mashhad, Iran [61]	2013–2014	CAP	≥16	4.2	-	-	Yes	1
Chengdu/Sichuan, China [62]	2013–2013	Respiratory illness	19–87	6.9	-	-	No	1
Foshan/Guangdong, China [63]	2013–2014	AE-COPD*	-	6.0	-	-	No	2
Chengdu/Sichuan, China [64]	2013–2014	Respiratory illness	>60	16.4	-	-	No	2
Beijing, China [65]	2013–2015	SARI	≥15	27.0	25.8	-	Yes	3
Haidlan/Beijing, China [66]	2013–2015	CAP	16–98	22.6	-	32.1	No	3

Location	Years	Case definition	Age range	% Influenza positive, all ages	% Influenza positive, 18–64 years	% Influenza positive, ≥65 years	PCR testing	Quality Score [1]
Urumchi/Xinjiang, China [67]	2013–2015	ALRI	18–84	14.2	-	-	No	2
Luzhou/Sichuan, China [68]	2014–2015	Respiratory illness	≥60	13.7	-	-	No	2
Chengdu/Sichuan, China [69]	2014–2015	Respiratory illness	≥16	20.6	21.5	18.6	No	2
Taizhou/Jiangsu, China [70]	2014–2015	FARI	-	11.3	-	-	Yes	1
Wuzhou/Guangxi, China [71]	2014–2015	ARI	15–93	2.7	3.0	2.6	No	2
Guangdong/Guangzhou, China [72]	2014–2015	SARI	≥20	19.4	21.3	14.3	Yes	1
Bozhou/Anhui, China [73]	2015–2016	AE-COPD*	25–94	75.3	-	-	No	2
Chaoyang/Beijing, China [74]	2015–2016	CAP	≥18	10.4	9.4	-	No	2
Urumchi/Xinjiang, China [75]	2015–2016	SARI	≥15	13.0	12.2	17.1	Yes	2
Fangshan/Beijing, China [76]	2015–2016	Influenza-related ICD-10 codes	≥20	8.6	6.0	8.8	Yes	2

*Study on a specific special population.

Abbreviations: ACS, acute coronary syndrome; AE, acute exacerbation; ARDS, acute respiratory distress syndrome; ARI, acute respiratory infection; CAP, community-acquired pneumonia; COPD, chronic obstructive pulmonary disease; FARI, febrile acute respiratory infection; ICU, intensive care unit; ILI, influenza-like illness; LRTI; lower respiratory tract infection; SARI, severe acute respiratory infection

References (S2 Table)

1. Wells G, B Shea B, O'Connell D, Peterson J , Welch V, Losos M, et al. The Newcastle-Ottawa Scale (NOS) for assessing the quality of nonrandomised studies in meta-analyses. [12/6/2019]; Available from: http://www.ohri.ca/programs/clinical_epidemiology/oxford.asp.
2. Jokinen C, Heiskanen L, Juvonen H, Kallinen S, Kleemola M, Koskela M, et al. Microbial etiology of community-acquired pneumonia in the adult population of 4 municipalities in eastern Finland. Clin Infect Dis. 2001 Apr 15;32(8):1141-54. 10.1086/319746.
3. Lieberman D, Schlaeffer F, Boldur I, Lieberman D, Horowitz S, Friedman MG, et al. Multiple pathogens in adult patients admitted with community-acquired pneumonia: a one year prospective study of 346 consecutive patients. Thorax. 1996 Feb;51(2):179-84.
4. Torres A, Dorca J, Zalacain R, Bello S, El-Ebiary M, Molinos L, et al. Community-acquired pneumonia in chronic obstructive pulmonary disease: a Spanish multicenter study. Am J Respir Crit Care Med. 1996 Nov;154(5):1456-61. 10.1164/ajrccm.154.5.8912764.
5. Teichtahl H, Buckmaster N, Pertnikovs E. The incidence of respiratory tract infection in adults requiring hospitalization for asthma. Chest. 1997 Sep;112(3):591-6.
6. Atmar RL, Piedra PA, Patel SM, Greenberg SB, Couch RB, Glezen WP. Picornavirus, the most common respiratory virus causing infection among patients of all ages hospitalized with acute respiratory illness. Journal of clinical microbiology. 2012 Feb;50(2):506-8. 10.1128/jcm.05999-11.
7. Scott JA, Hall AJ, Muyodi C, Lowe B, Ross M, Chohan B, et al. Aetiology, outcome, and risk factors for mortality among adults with acute pneumonia in Kenya. Lancet (London, England). 2000 Apr 8;355(9211):1225-30.
8. Ishida T HT, Arita M, Ito I, Osawa M. . Etiology of CAP in hospitalized patients, 3-year prospective study in Japan. Chest. 1998;114:1588-93.
9. Goh SK, Johan A, Cheong TH, Wang YT. A prospective study of infections with atypical pneumonia organisms in acute exacerbations of chronic bronchitis. Annals of the Academy of Medicine, Singapore. 1999 Jul;28(4):476-80.
10. Socan M, Marinic-Fiser N, Kraigher A, Kotnik A, Logar M. Microbial aetiology of community-acquired pneumonia in hospitalised patients. Eur J Clin Microbiol Infect Dis. 1999 Nov;18(11):777-82.
11. de Roux A, Marcos MA, Garcia E, Mensa J, Ewig S, Lode H, et al. Viral community-acquired pneumonia in nonimmunocompromised adults. Chest. 2004 Apr;125(4):1343-51.
12. Lieberman D, Lieberman D, Gelfer Y, Varshavsky R, Dvoskin B, Leinonen M, et al. Pneumonic vs nonpneumonic acute exacerbations of COPD. Chest. 2002 Oct;122(4):1264-70.
13. McNeill KM, Vaughn BL, Brundage MB, Li Y, Poropatich RK, Gaydos JC. Clinical presentations for influenza and influenza-like illness in young, immunized soldiers. Military medicine. 2005 Jan;170(1):94-7.
14. Zhang Zhonglu HXi. Clinical study on community- acquired adult respiratory tract infection of influenza virus A. ZhongGuo shiyong Neike Zazhi. 2003.
15. Lim WS, Macfarlane JT, Boswell TC, Harrison TG, Rose D, Leinonen M, et al. Study of community acquired pneumonia aetiology (SCAPA) in adults admitted to hospital: implications for management guidelines. Thorax. 2001 Apr;56(4):296-301.
16. Rohde G, Wiethege A, Borg I, Kauth M, Bauer TT, Gillissen A, et al. Respiratory viruses in exacerbations of chronic obstructive pulmonary disease requiring hospitalisation: a case-control study. Thorax. 2003 Jan;58(1):37-42.
17. Braun JJ, de Graaff CS, de Goey J, Zwinderman AH, Petit PL. [Community-acquired pneumonia: pathogens and course in patients admitted to a general hospital]. Nederlands tijdschrift voor geneeskunde. 2004 Apr 24;148(17):836-40.
18. Diederen BM, Van Der Eerden MM, Vlaspolder F, Boersma WG, Kluytmans JA, Peeters MF. Detection of respiratory viruses and Legionella spp. by real-time polymerase chain reaction in patients with community acquired pneumonia. Scand J Infect Dis. 2009;41(1):45-50. 10.1080/00365540802448799.
19. Caberlotto OJ, Cadario ME, Garay JE, Copacastro CA, Cabot A, Savy VL. Community-acquired pneumonia in patients from two different hospitals. Medicina. 2003;63(1):1-8.
20. Kobashi Y, Okimoto N, Matsushima T, Soejima R. Clinical analysis of community-acquired pneumonia in the elderly. Intern Med. 2001 Aug;40(8):703-7.
21. Jennings LC, Anderson TP, Beynon KA, Chua A, Laing RT, Werno AM, et al. Incidence and characteristics of viral community-acquired pneumonia in adults. Thorax. 2008 Jan;63(1):42-8. 10.1136/thx.2006.075077.

22. Hohenthal U, Vainionpaa R, Meurman O, Vahtera A, Katiskalahti T, Nikoskelainen J, et al. Aetiological diagnosis of community acquired pneumonia: utility of rapid microbiological methods with respect to disease severity. Scand J Infect Dis. 2008;40(2):131-8. 10.1080/00365540701534525.
23. Behbehani N, Mahmood A, Mokaddas EM, Bittar Z, Jayakrishnan B, Khadadah M, et al. Significance of atypical pathogens among community-acquired pneumonia adult patients admitted to hospital in Kuwait. Med Princ Pract. 2005 Jul-Aug;14(4):235-40. 10.1159/000085741.
24. Templeton KE, Scheltinga SA, van den Eeden WC, Graffelman AW, van den Broek PJ, Claas EC. Improved diagnosis of the etiology of community-acquired pneumonia with real-time polymerase chain reaction. Clin Infect Dis. 2005 Aug 1;41(3):345-51. 10.1086/431588.
25. Cameron RJ, de Wit D, Welsh TN, Ferguson J, Grissell TV, Rye PJ. Virus infection in exacerbations of chronic obstructive pulmonary disease requiring ventilation. Intensive care medicine. 2006 Jul;32(7):1022-9. 10.1007/s00134-006-0202-x.
26. Suttinont C, Losuwanaluk K, Niwatayakul K, Hoontrakul S, Intaranongpai W, Silpasakorn S, et al. Causes of acute, undifferentiated, febrile illness in rural Thailand: results of a prospective observational study. Annals of tropical medicine and parasitology. 2006 Jun;100(4):363-70. 10.1179/136485906x112158.
27. Oosterheert JJ, van Loon AM, Schuurman R, Hoepelman AI, Hak E, Thijsen S, et al. Impact of rapid detection of viral and atypical bacterial pathogens by real-time polymerase chain reaction for patients with lower respiratory tract infection. Clin Infect Dis. 2005 Nov 15;41(10):1438-44. 10.1086/497134.
28. Díaz F A, Couble P B, Fuentes L G, Uribe S. M R, Mercado M G, Soza G A, et al. Etiología de la neumonía adquirida en la comunidad en adultos hospitalizados en Santiago, Chile: implicancias para las guías clínicas. Rev chil enferm respir. 2005;21(1):23-32.
29. Angeles Marcos M, Camps M, Pumarola T, Antonio Martinez J, Martinez E, Mensa J, et al. The role of viruses in the aetiology of community-acquired pneumonia in adults. Antiviral therapy. 2006;11(3):351-9.
30. Daubin C, Parienti JJ, Vincent S, Vabret A, du Cheyron D, Ramakers M, et al. Epidemiology and clinical outcome of virus-positive respiratory samples in ventilated patients: a prospective cohort study. Critical care (London, England). 2006;10(5):R142. 10.1186/cc5059.
31. Muller A, Klinkenberg D, Vehreschild J, Cornely O, Tillmann RL, Franzen C, et al. Low prevalence of human metapneumovirus and human bocavirus in adult immunocompromised high risk patients suspected to suffer from Pneumocystis pneumonia. The Journal of infection. 2009 Mar;58(3):227-31. 10.1016/j.jinf.2009.01.004.
32. Hutchinson AF, Ghimire AK, Thompson MA, Black JF, Brand CA, Lowe AJ, et al. A community-based, time-matched, case-control study of respiratory viruses and exacerbations of COPD. Respiratory medicine. 2007 Dec;101(12):2472-81. 10.1016/j.rmed.2007.07.015.
33. Zhou YiPing LX, Chen XiaoKe, Yang LaiZhi, Liu Hui and Yu HaiQiong. Viral etiology of acute lower respiratory infection in adult inpatients. [Chinese]. Chinese Journal of Respiratory and Critical Care Medicine. 2010.
34. Johansson N, Kalin M, Tiveljung-Lindell A, Giske CG, Hedlund J. Etiology of community-acquired pneumonia: increased microbiological yield with new diagnostic methods. Clin Infect Dis. 2010 Jan 15;50(2):202-9. 10.1086/648678.
35. Ko FW, Ip M, Chan PK, Fok JP, Chan MC, Ngai JC, et al. A 1-year prospective study of the infectious etiology in patients hospitalized with acute exacerbations of COPD. Chest. 2007 Jan;131(1):44-52. 10.1378/chest.06-1355.
36. Minosse C, Selleri M, Zaniratti MS, Cappiello G, Longo R, Schifano E, et al. Frequency of detection of respiratory viruses in the lower respiratory tract of hospitalized adults. Journal of clinical virology : the official publication of the Pan American Society for Clinical Virology. 2008 Jun;42(2):215-20. 10.1016/j.jcv.2008.01.012.
37. Johnstone J, Majumdar SR, Fox JD, Marrie TJ. Viral infection in adults hospitalized with community-acquired pneumonia: prevalence, pathogens, and presentation. Chest. 2008 Dec;134(6):1141-8. 10.1378/chest.08-0888.
38. Rioseco ZM, Riquelme OR, Riquelme OM, Inzunza PC, Oyarzun GP, Aguero OY, et al. [Viral etiology of community acquired pneumonia among adults admitted to a general hospital]. Revista medica de Chile. 2012 Aug;140(8):984-9. 10.4067/s0034-98872012000800003.
39. Sumino KC, Walter MJ, Mikols CL, Thompson SA, Gaudreault-Keener M, Arens MQ, et al. Detection of respiratory viruses and the associated chemokine responses in serious acute respiratory illness. Thorax. 2010 Jul;65(7):639-44. 10.1136/thx.2009.132480.
40. Luchsinger V, Prades Y, Ruiz M, Pizarro R, Rossi P, Lizama L, et al. Comparison of Luminex xTAG(R) RVP fast assay and real time RT-PCR for the detection of respiratory viruses in adults with community-acquired pneumonia. J Med Virol. 2016 Jul;88(7):1173-9. 10.1002/jmv.24463.

41. Mermond S, Berlioz-Arthaud A, Estivals M, Baumann F, Levenes H, Martin PM. Aetiology of community-acquired pneumonia in hospitalized adult patients in New Caledonia. Trop Med Int Health. 2010 Dec;15(12):1517-24. 10.1111/j.1365-3156.2010.02653.x.
42. Kuanprasert S, Apichartpikul N, Chuenkitmongkol S, Wiangosot W, Topaiboon P, Sukonthasarn A. Evidence of influenza or influenza-like-illness preceding acute coronary syndrome. The Southeast Asian journal of tropical medicine and public health. 2008 Nov;39(6):1040-4.
43. Hara K, Yahara K, Gotoh K, Nakazono Y, Kashiwagi T, Imamura Y, et al. Clinical study concerning the relationship between community-acquired pneumonia and viral infection in northern Thailand. Intern Med. 2011;50(9):991-8.
44. Kherad O, Kaiser L, Bridevaux PO, Sarasin F, Thomas Y, Janssens JP, et al. Upper-respiratory viral infection, biomarkers, and COPD exacerbations. Chest. 2010 Oct;138(4):896-904. 10.1378/chest.09-2225.
45. ZHOU Yiping LXn, CHEN Xiaoke, et al. Study on the relationship between respiratory viruses and lower respiratory tract infection in adult patients. Chinese Journal of Respiratory and Critical Care Medicine. 2010.
46. Gambarino S, Mantovani S, Astegiano S, Libertucci D, Solidoro P, Baldi S, et al. Lower respiratory tract viral infections in hospitalized adult patients. Minerva medica. 2009 Oct;100(5):349-55.
47. Kwak HJ, Park DW, Kim JE, Park MK, Koo GW, Park TS, et al. Prevalence and Risk Factors of Respiratory Viral Infections in Exacerbations of Chronic Obstructive Pulmonary Disease. The Tohoku journal of experimental medicine. 2016 Oct;240(2):131-9. 10.1620/tjem.240.131.
48. Prasetyo AA, Desyardi MN, Tanamas J, Suradi, Reviono, Harsini, et al. Respiratory viruses and torque teno virus in adults with acute respiratory infections. Intervirology. 2015;58(1):57-68. 10.1159/000369211.
49. Jain S, Self WH, Wunderink RG, Fakhran S, Balk R, Bramley AM, et al. Community-Acquired Pneumonia Requiring Hospitalization among U.S. Adults. The New England journal of medicine. 2015 Jul 30;373(5):415-27. 10.1056/NEJMoa1500245.
50. Kim HC, Choi SH, Huh JW, Sung H, Hong SB, Lim CM, et al. Different pattern of viral infections and clinical outcomes in patient with acute exacerbation of chronic obstructive pulmonary disease and chronic obstructive pulmonary disease with pneumonia. J Med Virol. 2016 Dec;88(12):2092-9. 10.1002/jmv.24577.
51. Koul PA, Khan UH, Asad R, Yousuf R, Broor S, Lal RB, et al. Contribution of influenza to acute exacerbations of chronic obstructive pulmonary disease in Kashmir, India, 2010-2012. Influenza and other respiratory viruses. 2015 Jan;9(1):40-2. 10.1111/irv.12291.
52. 蒋小娟 赖a刘. 甘肃省2010-2013年942例下呼吸道感染病例病毒病原特征分析. 中华疾病控制杂志(07): 659-662. 2015(7): 659-62. [Jiang Xiaojuan, Lai Liu, et al. Analysis of viral characteristics of 942 cases of lower respiratory tract infection in Gansu Province from 2010 to 2013. Chinese Journal of Disease Control (07): 659-662 2015: 659-62.]
53. 李兵 李a陈. 支气管哮喘患者流感病毒检测的临床研究. 中华肺部疾病杂志(电子版)(02): 192-193. 2014(2):192-3. [Li Bing Li a Chen. Clinical study on detection of influenza virus in patients with bronchial asthma. Chinese Journal of Lung Diseases (Electronic Edition) (02): 192-193 2014: 192-3.]
54. Litwin CM, Bosley JG. Seasonality and prevalence of respiratory pathogens detected by multiplex PCR at a tertiary care medical center. Archives of virology. 2014 Jan;159(1):65-72. 10.1007/s00705-013-1794-4.
55. Bonizzoli M, Arvia R, di Valvasone S, Liotta F, Zakrzewska K, Azzi A, et al. Human herpesviruses respiratory infections in patients with acute respiratory distress (ARDS). Medical microbiology and immunology. 2016 Aug;205(4):371-9. 10.1007/s00430-016-0456-z.
56. 薛晓婕 程a. 老年呼吸道感染患者9项呼吸道病原体感染状况分析. 实用老年医学(05): 409-411. 2015(5):409-11. [Xue Xiaojie Cheng a. Analysis of 9 respiratory pathogen infections in elderly patients with respiratory infections. Practical Geriatrics (05): 409-411 2015: 409-11.]
57. Remolina YA, Ulloa MM, Vargas H, Diaz L, Gomez SL, Saavedra A, et al. Viral Infection in Adults with Severe Acute Respiratory Infection in Colombia. PLoS One. 2015;10(11):e0143152. 10.1371/journal.pone.0143152.
58. Gadsby NJ, Russell CD, McHugh MP, Mark H, Conway Morris A, Laurenson IF, et al. Comprehensive Molecular Testing for Respiratory Pathogens in Community-Acquired Pneumonia. Clin Infect Dis. 2016 Apr 1;62(7):817-23. 10.1093/cid/civ1214.
59. Refaey S, Amin M, Labib M, Kandeel A. Influenza virus positivity and circulating subtypes among cases of influenza-like illness and severe acute respiratory infection, Egypt, 2012-2015. Eastern Mediterranean health journal = La revue de sante de la Mediterranee orientale = al-Majallah al-sihhiyah li-sharq al-mutawassit. 2016 Oct 2;22(7):527-36.

60. Refaey S, Hassan M, Mansour A, Kandeel A. Incidence of influenza virus-associated severe acute respiratory infection in Damanhour district, Egypt, 2013. Eastern Mediterranean health journal = La revue de sante de la Mediterranee orientale = al-Majallah al-sihhiyah li-sharq al-mutawassit. 2016 Oct 2;22(7):503-12.

61. Naderi H, Sheybani F, Sarvghad M, Meshkat Z, Jabbari Nooghabi M. Etiological Diagnosis of Community-Acquired Pneumonia in Adult Patients: A Prospective Hospital-Based Study in Mashhad, Iran. Jundishapur J Microbiol. 2015 Aug;8(8):e22780. 10.5812/jjm.22780.

62. 陈华根 刘, 黄学斌 and 宋强. 呼吸道疾病非典型病原体检测结果分析. 临床血液学杂志(输血与检验)(06): 1071-1073. 2014(6):1071-3. [Chen Huagen Liu, Huang Xuebin and Song Qiang. Analysis of the detection results of atypical pathogens of respiratory diseases. Journal of Clinical Hematology (Blood Transfusion and Test) (06): 1071-1073 2014: 1071-3.]

63. 朱翠珍 刘, 温新云 and 郑雪婷. 慢性阻塞性肺疾病急性加重期病原学分析. 国际检验医学杂志(15): 2159-2161. 2016(15):2159-61. [Zhu Cuizhen Liu, Wen Xinyun and Zheng Xueting. Pathogenic analysis of acute exacerbation of chronic obstructive pulmonary disease. International Journal of Laboratory Medicine (15): 2159-2161 2016: 2159-61.]

64. 陈华根 刘a. 老年呼吸道疾病患者呼吸道非典型病原体IgM抗体检测结果分析. 检验医学与临床(02): 209-210+212. 2015(2): 209-10+12. [Chen Huagen Liu a. Analysis of IgM antibody detection results of respiratory atypical pathogens in elderly patients with respiratory diseases. Laboratory Medicine and Clinical (02): 209-210 + 212 2015: 209-10 + 12.]

65. 赵小娟 于, 刘建华, 付迎新, 马秀红, 杨剑, 刘利英, 李超 and 王保东. 北京市怀柔区2013-2015年流感住院患者严重急性呼吸道感染流行病学与临床特征分析. 中国预防医学杂志(02): 124-128. 2017(2): 124-8. [Zhao Xiaojuan Yu, Liu Jianhua, Fu Yingxin, Ma Xiuhong, Yang Jian, Liu Liying, Li Chao and Wang Baodong. Epidemiological and clinical characteristics analysis of severe acute respiratory infections in hospitalized influenza patients in Huairou District, Beijing, 2013-2015. Chinese Journal of Preventive Medicine (02): 124-128 2017: 124-8.]

66. 李月越 王, 陈杭薇 and 赵雅辉. 病毒及非典型病原体致社区获得性肺炎住院患者的临床分析. 临床肺科杂志(02): 279-283. 2017(2):279-83. [Li Yueyue Wang, Chen Hangwei and Zhao Yahui. Clinical analysis of hospitalized patients with community acquired pneumonia caused by viruses and atypical pathogens. Journal of Clinical Pulmonary Medicine (02): 279-283 2017: 279-83.]

67. 凌敏 吴a. 成人急性下呼吸道感染中直接免疫荧光法病毒抗原检测结果分析. 新疆医学(02): 166-169. 2016(2):166-9. [Ling Min Wu a. Analysis of the results of direct immunofluorescence virus antigen detection in adult acute lower respiratory tract infections. Xinjiang Medical Journal (02): 166-169 2016: 166-9.]

68. 涂业桃 姜a舒. 935例老年呼吸道疾病患者非典型病原体检测结果分析. 重庆医学(03): 382-384. 2017(3):382-4. [Ding Weitan, Li Xuemei, Jiang Changli, Cai Dixin, Zhang Yue, Wang Lan and Pan Xinghua. Analysis of pathogen detection results in adult patients with acute respira[Tu Yetao Jiang Ashu. Analysis of the results of atypical pathogen detection in 935 elderly patients with respiratory diseases. Chongqing Medical (03): 382-384 2017: 382-4.]

69. 丁伟 谭, 李雪梅, 姜昌丽, 蔡第心, 张悦, 王澜 and 潘兴华. 急性呼吸道感染成年患者病原体检测结果分析. 国际检验医学杂志(05): 577-578+581. 2017(5): 577-8+81. [Ding Weitan, Li Xuemei, Jiang Changli, Cai Dixin, Zhang Yue, Wang Lan and Pan Xinghua. Analysis of pathogen detection results in adult patients with acute respiratory infections. International Journal of Laboratory Medicine (05): 577-578 + 581 2017: 577-8 + 81 .]

70. 朱小华 许a李. 2014-2015年泰州地区急性呼吸道感染病毒病原学研究. 临床肺科杂志(10): 1912-1915. 2016(10):1912-5. [Zhu Xiaohua, Xu a Li. Aetiology of acute respiratory infection virus in Taizhou region from 2014 to 2015. Journal of Clinical Pulmonary Medicine (10): 1912-1915 2016: 1912-5.]

71. 梁世廉 黄, 黄献欢, 李迅维, 龙何英, 赵献连, 白敏, 陈妤 and 黄文俪. 梧州地区住院患者呼吸道病毒及非典型病原体的抗体检测分析. 临床肺科杂志(12): 2277-2280. 2016(12): 2277-80. [Liang Shilian Huang, Huang Xianhuan, Li Xunwei, Long Heying, Zhao Xianlian, Bai Min, Chen Yi, and Huang Wenyi. Detection and analysis of antibodies against respiratory viruses and atypical pathogens in hospitalized patients in Wuzhou. Journal of Clinical Pulmonary Medicine (12): 2277-2280 2016 : 2277-80.]

72. 曹蓝 陈, 鲁恩洁, 陆剑云, 陈宗遒, 马钰, 刘艳慧, 袁润余, 狄飚 and 李魁彪. 广州市2014—2015年严重急性呼吸道感染病例的流感病毒流行特征分析. 华南预防医学(03): 240-243. 2017(3):240-3. [Cao Lanchen, Lu Enjie, Lu

Jianyun, Chen Zongxi, Ma Yu, Liu Yanhui, Yuan Runyu, Di Jun and Li Kuibiao. Analysis of the epidemic characteristics of influenza virus in cases of severe acute respiratory infection in Guangzhou from 2014 to 2015. South China Preventive Medicine (03) : 240-243 2017: 240-3.]

73. 孙峰 张, 张永娟, 梅倩, 昝丽娜 and 刘广印. 慢性阻塞性肺疾病急性加重患者中病原体的检测分析. 临床输血与检验(02): 162-166. 2017(2):162-6. [Sun Feng Zhang, Zhang Yongjuan, Mei Qian, Tong Lina and Liu Guangyin. Detection and analysis of pathogens in patients with acute exacerbation of chronic obstructive pulmonary disease. Clinical Blood Transfusion and Testing (02): 162-166 2017: 162-6.]

74. 延峰 王a黄. 2015-2016年社区获得性肺炎患者非典型病原菌分析. 山西医科大学学报(08): 823-826. 2017(8): 823-6. [Yan Feng Wang a Huang. Analysis of atypical pathogens in patients with community-acquired pneumonia from 2015 to 2016. Journal of Shanxi Medical University (08): 823-826 2017: 823-6.]

75. 赵俊 刘, 郜振国, 张璇, 刘红斌, 马合木提, 邓红 and 岳锡宏. 347例SARI病例15种常见呼吸道病原特征分析. 病毒学报(05): 761-766. 2017(5):761-6. [Zhao Jun Liu, Tong Zhenguo, Zhang Xuan, Liu Hongbin, Ma Hemuti, Deng Hong and Yue Xihong. Analysis of the characteristics of 15 common respiratory pathogens in 347 cases of SARI. Acta Virologica Sinica (05): 761-766 2017: 761-6.]

76. 石磊 黄, 刘静, 田竞, 李丽丽 and 张奕. 2015年4月-2016年4月北京市房山区流感相关住院病例的临床与病原学特征分析. 实用预防医学(09): 1051-1054. 2017(9): 1051-4. [Shi Leihuang, Liu Jing, Tian Jing, Li Lili and Zhang Yi. Analysis of clinical and etiological characteristics of flu-related hospitalized patients in Fangshan District, Beijing from April 2015 to April 2016. Practical Preventive Medicine (09): 1051- 1054 2017: 1051-4.]
