# Supplementary material for: Global burden of influenza-associated lower respiratory tract infections and hospitalizations among adults: A systematic review and meta-analysis
Source: PLoS Med. 2021 Mar 1;18(3):e1003550. doi: 10.1371/journal.pmed.1003550 (PMC7959367; doi:10.1371/journal.pmed.1003550)
Supplement: S3 Table — (PDF) [file pmed.1003550.s006.pdf]

Supplementary Table 3. Median number of specimens tested and percent positive for influenza, by age group, study design, and population among all data sources

	Number of data sources (n=112)	Median total number specimens tested	Interquartile Range (IQR)		Median percent positive	IQR		p-value
**All adults**	112	**361**	183	1137	**11%**	7%	19%	
**Age group**								
Adults <65 years*	50	**727**	319	1934	**15%**	9%	21%	0.28
Adults ≥65 years	48	**219**	90	667	**13%**	8%	17%	
**Data source**								
GRIPP Surveillance datasets	37	**1308**	565	3222	**15%**	10%	20%	0.011
Published papers	75	**242**	129	470	**10%**	6%	17%	
**Timeframe**								
Pre-2009 data only	45	**187**	119	296	**8%**	5%	16%	0.016
Post-2009 data only	61	**569**	328	1890	**14%**	8%	20%	
Pre- & post-2009 data	6	**2294**	1188	7487	**10%**	10%	15%	
**Diagnostic Test**								
RT-PCR only	40	**452**	204	1161	**14%**	8%	20%	0.58
Immunofluorescence only	12	**556**	397	1053	**9%**	6%	15%	
Serological assay only	13	**250**	135	338	**11%**	6%	16%	
Multiple tests, including RT-PCR	38	**455**	189	1755	**10%**	7%	17%	
Multiple tests, excluding RT-PCR	6	**121**	80	174	**14%**	7%	21%	
Other[†]	3	**340**	321	412	**9%**	9%	16%	
**Case definition**								
Acute respiratory infection (ARI)	7	**266**	164	312	**9%**	6%	13%	0.0014
Lower respiratory infection (LRI)	8	**850**	516	3697	**14%**	11%	15%	
Pneumonia	32	**189**	127	302	**7%**	6%	11%	
Severe acute respiratory infection (SARI)	35	**1308**	444	3213	**17%**	11%	20%	
Other[‡]	30	**412**	190	579	**10%**	5%	18%	
**Special population**[¶]								
Yes	20	**194**	102	394	**9%**	4%	19%	0.43
No	92	**462**	206	1384	**11%**	7%	19%	
**World Health Organization region**								
Africa	9	**1259**	411	1766	**7%**	6%	11%	0.071
Americas	18	**306**	164	1947	**9%**	6%	17%	

Eastern Mediterranean	8	**729**	220	1951	**20%**	17%	21%	
Europe	23	**187**	118	279	**8%**	3%	14%	
Southeast Asia	14	**714**	407	1770	**13%**	11%	16%	
Western Pacific	40	**470**	244	1015	**13%**	8%	19%	
**World Bank income level**								
Low	10	**1006**	530	1755	**12%**	7%	14%	0.035
Lower-middle	26	**825**	385	1758	**15%**	10%	19%	
Upper-middle	31	**483**	226	1342	**14%**	7%	20%	
High	45	**240**	124	328	**9%**	4%	16%	

*Gaps in age-specific estimates due to lack of outcome data stratified by age group.

† Includes rapid immunochromatographic assay (n=2), virus culture (n=1).

‡ Includes acute exacerbation of chronic obstructive pulmonary disease (COPD) (n=14), respiratory hospitalization (acute or non-acute) (n=10), acute exacerbation of asthma (n=3), acute febrile illness (n=2), intubation among intensive care unit (ICU) patients (n=1), acute respiratory distress syndrome (ARDS) (n=1), ICD codes consistent with influenza (n=1), and acute coronary syndrome with recent influenza-like illness (n=1).

¶ Includes studies of populations with COPD (n=10), hospitalised in the ICU only (n=3), immunocompromising conditions (n=1), asthma (n=1), heart disease (n=1), or in the military (n=1)
