# Supplementary material for: Global burden of influenza-associated lower respiratory tract infections and hospitalizations among adults: A systematic review and meta-analysis
Source: PLoS Med. 2021 Mar 1;18(3):e1003550. doi: 10.1371/journal.pmed.1003550 (PMC7959367; doi:10.1371/journal.pmed.1003550)
Supplement: S4 Table — (PDF) [file pmed.1003550.s007.pdf]

Supplementary Table 4. Regional estimates of influenza-associated lower respiratory tract infection (LRI) episodes and hospitalizations, by age group

	All-cause estimate* (95% CI) [thousands]	Influenza-associated events (95% CI) [thousands]	Rate per 100 000 population (95% CI)
**Africa**			
Hospitalized LRI episodes	11,050 (6,699—17,209)	1,560 (842—2,683)	326 (176—561)
LRI episodes	34,601 (28,763—41,050)	4,884 (3,050—6,966)	1,021 (637—14,56)
**Americas**			
Hospitalized LRI episodes	6,635 (4,495—9,314)	936 (550—1,485)	137 (80—217)
LRI episodes	23,406 (20,334—26,777)	3,304 (2,149—4,639)	483 (314—678)
**Eastern Mediterranean**			
Hospitalized LRI episodes	2,950 (1,761—4,671)	416 (224—718)	107 (57—184)
LRI episodes	20,996 (17,071—25,344)	2,964 (1,874—4,284)	760 (481—1,099)
**Europe**			
Hospitalized LRI episodes	8,512 (5,600—12,267)	1,201 (677—1,946)	170 (96—276)
LRI episodes	22,212 (18,989—25,797)	3,135 (2,025—4,415)	444 (287—626)
**Southeast Asia**			
Hospitalized LRI episodes	4,446 (2,706—6,889)	628 (349—1,070)	51 (28—87)
LRI episodes	98,555 (81,841—116,740)	13,911 (8,988—19,886)	1,134 (732—1,620)
**Western Pacific**			
Hospitalized LRI episodes	6,376 (4,389—8,830)	900 (531—1,444)	62 (37—100)
LRI episodes	27,186 (22,860—32,100)	3,837 (2,469—5,528)	266 (171—383)

*All-cause estimates are derived from Global Burden of Disease project, adapted for 2016, among individuals 20 years and older.
