# Supplementary material for: Global burden of influenza-associated lower respiratory tract infections and hospitalizations among adults: A systematic review and meta-analysis
Source: PLoS Med. 2021 Mar 1;18(3):e1003550. doi: 10.1371/journal.pmed.1003550 (PMC7959367; doi:10.1371/journal.pmed.1003550)
Supplement: S5 Table — (PDF) [file pmed.1003550.s008.pdf]

Supplementary Table 5. Sensitivity analyses

Model description	N (datasets)	Percent influenza positive	95% CI	I^2	Influenza-associated hospitalizations [thousands]	95% CI	% change in estimate
Base model	63	14.1%	12.1–16.5%	98%	5678	3205–9432	NA
Datasets using LRI case definition	7	13.4%	9.0–20.1%	87%	5404	1057–12349	-5%
Pre-2009 datasets	18	8.5%	5.4–12.7%	94%	3402	0–9221	-40%
Post-2009 datasets	49	15.4%	13.0–18.3%	98%	6206	3469–10326	9%
Pneumococcal conjugate vaccine (PCV) program present	26	13.4%	10.1–17.4%	96%	5384	2404–9841	-5%
No PCV program present	46	13.8%	11.6–16.4%	98%	5556	2726–9978	-2%
Published datasets	28	12.1%	9.2–15.7%	96%	4877	0–11241	-14%
Working group datasets	35	15.6%	13.0–18.9%	97%	6294	3548–10395	11%
All adults – Influenza A	51	10.6%	8.9-12.5%	98%	4264	2185–7353	NA
All adults – Influenza B	46	3.5%	2.8-4.3%	97%	1408	322–3034	NA
Adults 20-64 years – any influenza	41	16.5%	13.7-19.8%	97%	3464	1885–5978	NA
Adults ≥ 65 years – any influenza	38	14.7%	12.7-16.8%	97%	2831	1716–3969	NA
